# *In Vitro* Epithelial Organoid Generation Induced by Substrate Nanotopography

**DOI:** 10.1038/srep09293

**Published:** 2015-03-19

**Authors:** Yusheng Shen, Youmin Hou, Shuhuai Yao, Pingbo Huang, Levent Yobas

**Affiliations:** 1 Division of Biomedical Engineering, The Hong Kong University of Science & Technology, Hong Kong; 2 Department of Mechanical Engineering, The Hong Kong University of Science & Technology, Hong Kong; 3 Division of Life Science, The Hong Kong University of Science & Technology, Hong Kong; 4 The State Key Laboratory of Molecular Neuroscience, The Hong Kong University of Science & Technology, Hong Kong; 5 Department of Electronic & Computer Engineering, The Hong Kong University of Science & Technology, Hong Kong

## Abstract

The extracellular matrix (ECM) exhibits tissue-specific topography and composition and plays a crucial role in initiating the biochemical and biomechanical signaling required for organizing cells into distinct tissues during development. How single cells assemble into structures featuring specific shapes in response to external cues is poorly understood. We examined the effect of substrate nanotopography on the morphogenesis of several types of epithelial cells and found that in response to the topography, Calu-3 and MDCK-II cells formed organoids that closely resemble their morphology *in vivo*. This finding represents the first demonstration that substrate nanotopography, one of the first physical cues detected by cells, can by itself induce epithelial tissue-like organization. Our results provide insights, in terms of a new aspect of ECM topography, into the design of future tissue-engineering systems and the study of mechanosignaling in the epithelium during normal development and tumor progression.

The assembly of single epithelial cells into complex polarized tubule networks is vital for the functionality of many organs. This assembly requires the precise interpretation and incorporation of various external and internal signals that guide the acquisition of apicobasal polarity and the generation of a functional lumen by the cellular machinery, and this makes engineering such tissues extremely challenging^1^,^2^. Over the past 2 decades, methods of generating epithelial organoids have been developed and they mainly involve providing cells with a 3D culture environment in which chemical cues provided by ECM proteins induce rapid epithelial polarization and tissue organization^3^,^4^. Another critical cue that the ECM could present is a rich 3D surface topography at the nano and micro scales; this topography has been widely recognized to participate in controlling cellular functions including cell adhesion, migration, proliferation, and differentiation. However, very few studies have evaluated how surface topography affects epithelial tissue-like morphogenesis^2^,^5^,^6^,^7^,^8^. Among these studies, it has been found that confined microenvironment could promote lumen formation of epithelial and endothelial cells^9^,^10^ and nanotopography has been shown to have localized restrictive effects on cell spreading^11^,^12^. We hypothesized that epithelial organoid formation could be initiated by restricting cell spreading and migration by presenting cells with randomly aligned nano structures.

In this study, we fabricated randomly aligned nanograss substrates through a simple reactive-ion etching technique and demonstrated for the first time that human airway epithelial Calu-3 cells and MDCK-II cells formed tissue-like structures containing lumens in response to such surface topography. Our results clearly reveal the crucial role that ECM topography could play in dictating epithelial morphogenesis and cell fate.

## Results

### Calu-3 and MDCK-II cells response to nanograss in normal culture

**Figure 1:**
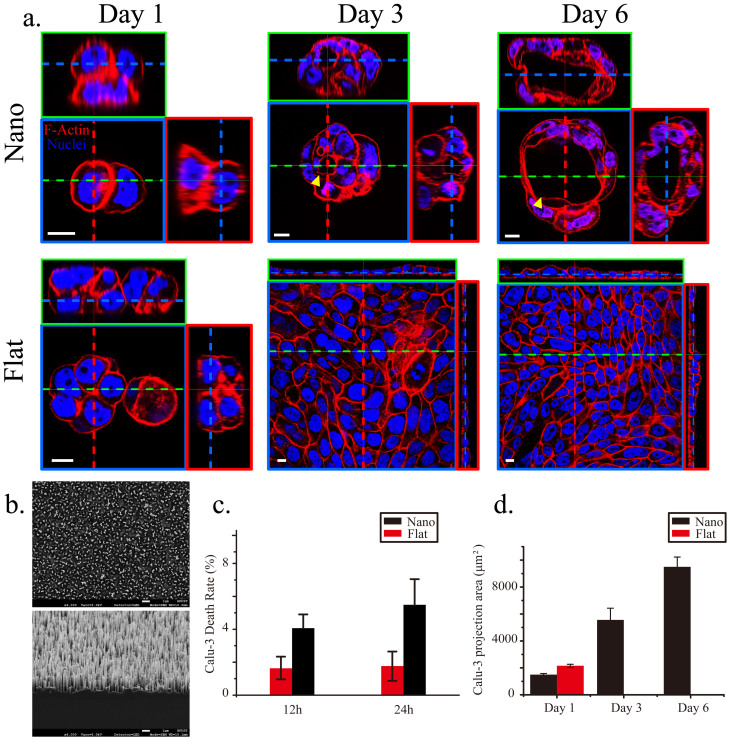
Calu-3 cells formed organoids on the nanograss. (a) Co-staining of F-actin (red) and the nuclei (blue) in Calu-3 cells cultured on nanograss-patterned and flat substrates from Day 1 to Day 6. In 3D images shown in this and in all other figures, blue dashed lines in the small green and red boxes indicate the position of the X-Y plane of focus in the blue box; the green and red dashed lines indicate the X-Z and Y-Z sections, shown in the green and red boxes, respectively. Yellow arrowheads indicate lumens. (b) SEM images of the nanograss-patterned silicon substrates (top, top view; bottom, side view). The nanograss is approximately 4 μm deep. (c) Calu-3-cell death rate after incubation for 12 and 24 h on the nanograss-patterned and flat substrates (n = 3 independent experiments, each with 5 populations of cells; *P* = 0.908 for 12 h and 0.391 for 24 h). (d) Statistics of Calu-3-cell colony-projection area after 1, 3, and 6 d of culture on the nanograss-patterned and flat substrates. No significant difference was observed on Day 1 (n = 3 independent experiments, each with 10 populations of cell colonies; *P* = 0.0565). Calu-3 cells became confluent after 3 d of culture on the flat substrates, and thus the projection area of separated colonies could not be quantified. Scale bars, 10 μm (a) and 1 μm (b).

**Figure 2:**
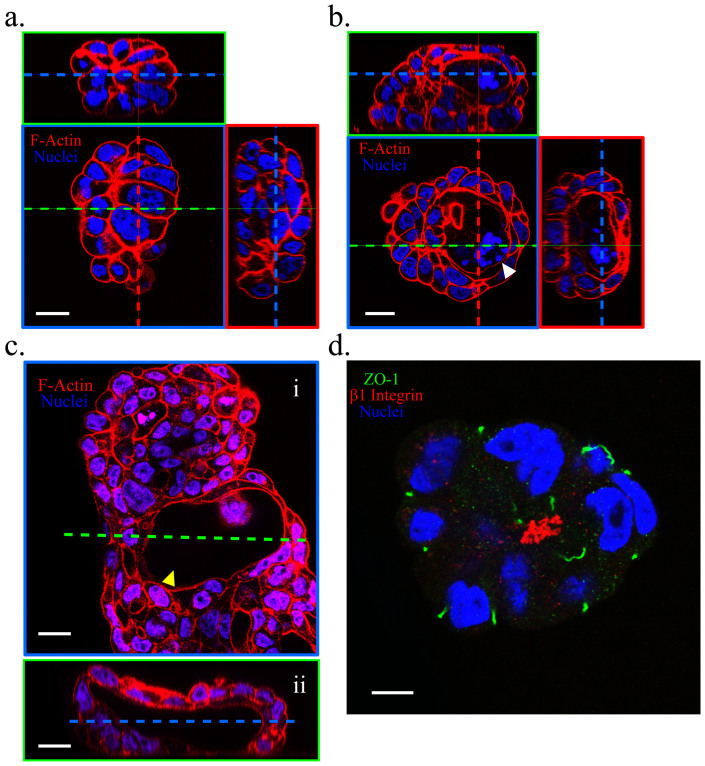
Calu-3 organoids assumed a reversed polarity (inside-out; basal surface facing the central lumen). On Day 6 of culture, spheroids lacking a lumen (a), containing a developing lumen (b) (white arrowhead), and containing a fully formed lumen (c) (yellow arrowhead; the longest gap of the lumen reached 100 μm) were observed when cultures were co-stained for F-actin (red) and the nuclei (blue). (d) Co-immunostaining of ZO-1 (an apical-domain marker, green), β1 integrin (a basal-domain marker, red), and the nuclei (blue) showing the polarity of one Calu-3 organoid. Scale bars, 20 μm (a, b, c) and 10 μm (d).

Using scanning electron microscopy (SEM), we verified the geometry and dimensions of randomly aligned nanograss featuring a high aspect ratio (>10) (Fig. 1). We developed this design based on 2 considerations: (1) The effect of contact guidance on epithelial morphogenesis can be minimized when nanospikes are arranged stochastically and isotropically in order to locally restrict cell spreading, migration, and proliferation along the horizontal plane^2^,^5^,^6^,^7^,^10^; (2) Intense, dynamic, and reciprocal interactions between cells and topographic patterns can be achieved when the accessibility and flexibility of nanograss are controlled by modifying nanograss density and aspect ratio^11^,^12^,^13^,^14^. First, we assessed the viability of single Calu-3 cells exposed to the nanograss pattern or to a flat topography after culturing the cells for 12 and 24 h (Fig. 1c). The death rate of Calu-3 cells incubated on the nanograss-patterned substrate was <10% and was comparable to that of cells cultured on the flat substrate, indicating that the flat and nanotopographic substrates were equally suitable for the initial growth and survival of Calu-3 cells. Subsequently, we examined the morphology of Calu-3 cells on Days 1, 3, and 6 (Fig. 1a). After culturing for 1 d, most cells appeared to have developed into separated small spheroids on both the nanograss-patterned and flat substrates, but the mean projection area of the cell colonies on the flat surface was moderately larger than that on the nanograss surface (*P* = 0.0565), suggesting that the nanotopographic feature might suppress the spreading and migration of Calu-3 cells (Fig. 1d). This local, horizontal restriction became clearer as the cells were cultured longer: on the flat substrate, single cell colonies eventually merged with neighboring colonies to form subconfluent (on Day 3) or confluent (on Day 6) monolayers (Fig. 1d); by contrast, the merging of colonies was largely inhibited on the nanograss substrate, and the increase in colony size led to a vertical growth of cells and the formation of tissue-like spheroids (Fig. 1a). Intriguingly, a lumen formed *de novo* and expanded within the cell aggregates starting on Day 3 (Fig. 1a and Supplementary Fig. S1) and its morphology closely resembled that of acini *in vivo*. Close inspection of the spheroids revealed 3 forms on Day 6: spheroids lacking a lumen, spheroids featuring a developing lumen, and spheroids containing a fully formed lumen (up to 100 μm in diameter); these presumably reflect distinct stages of the development of cell aggregates into organoids (Fig. 2). Notably, fragmentation of the nuclear material (Fig. 2b) and the presence of active caspase-3 (Supplementary Fig. S2) – an apoptosis marker^15^ were widely observed within developing lumens, which indicated that the lumen formed as a result of apoptosis and clearance of inner cells, one of the two predominant mechanisms of *de novo* lumen formation^1^,^16^. Examining the co-staining of ZO-1 (an apical-domain marker), β1 integrin (a basal-domain maker), and the nuclei showed that the cyst-like structures featured a reversed polarity, with the basal side facing the lumen (Fig. 2d), which is the same polarity observed in suspension cultures^17^. Collectively, our data suggest that Calu-3 epithelial cells can form organoids containing lumens in response to substrate nanotopography.

**Figure 3:**
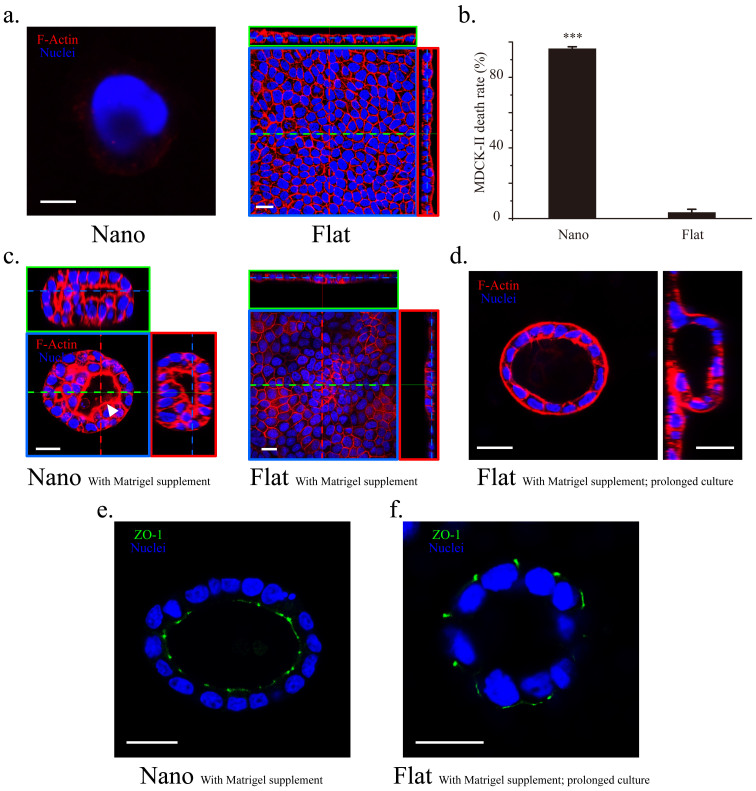
MDCK-II cells responded to nanotopography distinctly from Calu-3 cells. (a) Co-staining of F-actin (red) and the nuclei (blue) showed that a single MDCK-II cell died after 1 d when cultured on the nanograss-patterned substrate (left, F-actin is almost undetectable); by contrast, MDCK-II cells formed a confluent monolayer on the flat substrate after 3 d in culture (right). (b) Death rate of adherent MDCK-II cells after incubation for 12 h on the nanograss-patterned and flat substrates (n = 3 independent experiments, each with 5 populations of cells; ***, *P* = 9.92E−5 compared to the flat substrate). (c) MDCK-II cells formed spheroids with lumens on the nanograss while maintained as monolayer on the flat substrates with 2% Matrigel supplement in the culture medium on Day 3. The lumen (white arrow head) is partially filled with fragmented nuclear material. (d) On Day 6, prolonged culture of MDCK-II cells on the flat substrates with Matrigel supplement generated lumens by buckling and wrapping of the cell sheet (left, top view; right, side view). (e, f) The lumens generated on the nanograss and the flat substrates featured a normal and a reversed polarity, respectively. Scale bars, (a): 5 μm (left) and 20 μm (right); 20 μm (c–f).

Next, we investigated whether substrate topography also induces other epithelial cell types to form organoids. When MDCK-II cells (a common epithelial cell model) were exposed to the same nanotopographic feature for 12 h, massive cell death occurred (Fig. 3). Positive staining of propidium iodide (PI) in those dead cells suggests that they underwent necrosis (Fig. 3b). In the dead cells, F-actin was almost undetectable, indicating loss of the cytoskeleton and presumably an intact membrane (Fig. 3a). Thus, we determined whether a difference in the interaction between the substrate and the two cell types underlies the death of MDCK-II cells. SEM analysis of single dead MDCK-II cells revealed extensively intimate interaction between the cells and the substrate, which was indicated by the substantial bending of the nanograss toward the cells (Supplementary Fig. S3). Although similar nanograss engagement was observed in the case of single Calu-3 cells (Supplementary Fig. S4), cell death did not occur. These results excluded the possibility that MDCK-II cells were killed by penetration of nanospikes. We also tested the response of 3 other epithelial cell types to substrate nanotopography. Both BEAS-2B and HCT116 cells encountered massive death on the nanograss (Supplementary Fig. S5). BEAS-2B and HCT116 cells are derived from normal bronchial epithelium and colorectal carcinomas, respectively. Cell-type-dependent cell death in response to surface topography has been reported previously, but the underlying molecular mechanism remains unexplored^18^,^19^,^20^,^21^. Interestingly, T84 cells derived from colon carcinomas, formed monolayer colonies and confluent monolayer on the nanograss and flat substrates, respectively (Supplementary Fig. S5 and see discussion).

### Calu-3 and MDCK-II cells response to nanograss in “in-3D” culture

In order to evaluate the effect of nanotopography on the morphogenesis of MDCK-II cells, an “in-3D” culture method^9^, in which the culture medium was supplemented with 2% Matrigel, was tried to rescue MDCK-II cells on the nanograss. Matrigel, an extract of the copious ECM secreted by Englebreth-Holm-Swarm tumors, is primarily composed of laminin, collagen IV, and entactin^22^. Laminin reportedly play an essential role, as both a physical scaffold and a biochemical and biomechanical inducer, in the polarization and tissue-like morphogenesis of epithelial cells^23^,^24^,^25^,^26^. On Day 3, interestingly, a limited number of isolated MDCK-II colonies survived and formed lumens on the nanograss while on the flat substrates, MDCK-II cells only formed a confluent monolayer (Fig. 3c) and lumens can only develop with a prolonged cell culture on Day 6 (Fig. 3d). Importantly, further ZO-1 staining showed that the lumens formed on the nanograss and flat substrates featured a normal and a reversed polarity, respectively (Fig. 3e, f). These results are consistent with our hypothesis and previous findings on “in-3D” culture of MDCK cells on laminin coated substrates^9^ and strongly imply that the restriction from the nanograss on the spreading, migration and proliferation of MDCK-II cells can promote early lumen generation from small cell colonies.

**Figure 4:**
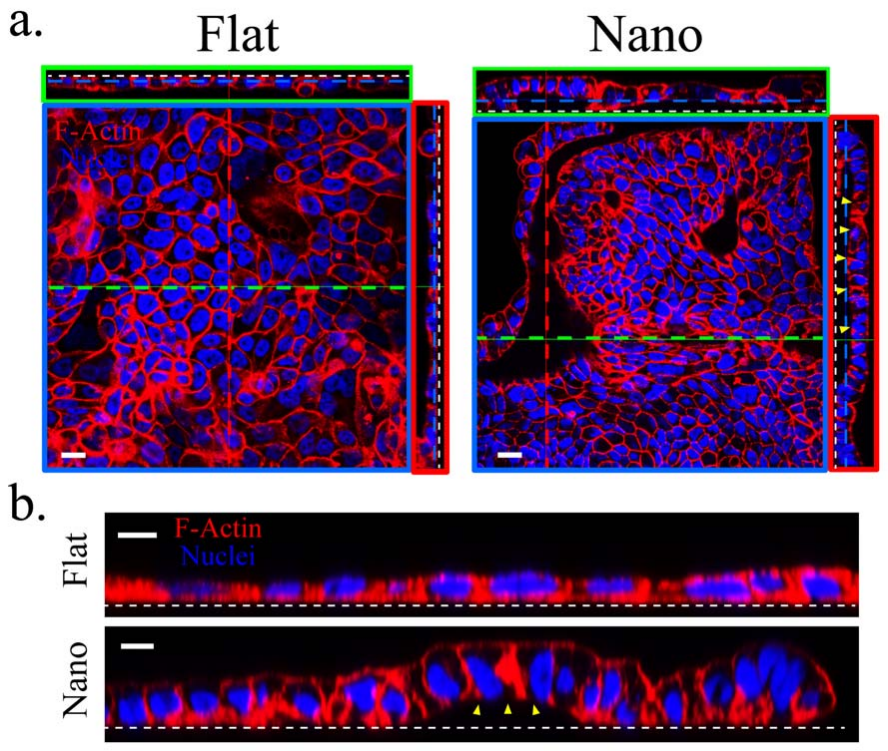
Calu-3 cells underwent different morphologic changes on the nanograss in the “in-3D” culture. (a) Calu-3 cells stabilized with monolayer morphology with extensive buckling on the nanograss while maintained as monolayer on the flat substrates on Day 15. Note that the gap (yellow arrowheads) between the basal surface of the Calu-3 cell sheet and the nanograss is about 10 μm after the folding. White dashed line indicates the cell-substrate interface. (b) The cell height of Calu-3 cells increased about two folds on the nanograss compared to that cultured on the flat substrates. Scale bars, 20 μm (a) and 10 μm (b).

Interestingly, the “in-3D” culture did not induce Calu-3 cells to develop lumens on the flat substrates even with a prolonged culture period but altered the response of Calu-3 cells on the nanograss (Fig. 4). On the nanograss, Calu-3 cells displayed various morphologies: monolayer, multilayer, spheroids, and even tubular structures on Day 6 (Supplementary Video S1), all of which finally stabilized as a monolayer with extensive buckling on Day 15 (Fig. 4a). Moreover, the cell height increased about two folds at the folded region of the cell sheet compared with that of those cultured on the flat substrate (Fig. 4b). The cell height increase and buckling of epithelial sheets closely resembled the cell behaviors during epithelial placodes formation, soon after which the placodes bends, invaginates, and forms lumen structures^27^,^28^.

### Filopodia and stress fiber formation in normal and “in-3D” culture

On the nanotopographic substrate, Calu-3 cells in normal culture used only filopodia-like structures to adhere to the nanograss and further extend the periphery of cell colonies, but these cells formed lamellipodia on the flat substrate on Day 1(Supplementary Fig. S6a). These results obtained using short-term cultures agree with the previous findings showing that cells grown on nanoneedles or nanorods only form filopodia^19^,^20^. On Day 3, at the center of cell colonies grown on flat substrates, F-actin exhibited a fiber-like distribution, which indicated the formation of mature stress fibers and focal adhesions; by comparison, at the center of colonies on the nanograss-patterned substrates, the distribution of F-actin was less discrete (Supplementary Fig. S6b). This difference in F-actin distribution was confirmed in culturing cells for prolonged periods (Supplementary Fig. S6c) and was also observed in T84 cells (Supplementary Fig. S5). By contrast, the introduction of Matrigel in the “in-3D” culture caused Calu-3 cells to form mature stress fibers on the nanograss (Supplementary Fig. S7).

## Discussion

We suggest that the inability of Calu-3 cells to form lamellipodia and mature focal adhesions on the nanograss in normal culture (Supplementary Fig. S6) might account for the suppression of the spreading and migration of Calu-3 cell colonies^29^,^30^ (Fig. 1d). Because of such a restriction along the horizontal plane, small piled-up of colonies cells might develop vertically into large spheroid structures containing lumens, and consequently failed to merge with other colonies to form a confluent monolayer. Importantly, the responses of HCT116 and T84 cells to the nanograss (Supplementary Figure S5) indicate that such spheroids formation of Calu-3 cells is probably not due to substrate/anchorage-independent growth of cancer cell lines. The observation that Calu-3 cells finally stabilized as monolayer morphology with prolong culture periods on the nanograss in the “in-3D” culture is somewhat surprising. One possibility is that the surface chemistry of the nanograss was modified by Matrigel coating during the “in-3D” culture and thus the restriction imposed by the nanograss was at least partially relaxed, as evidenced by the fact that Calu-3 cells regained the capability to form mature stress fibers on nanograss (Supplementary Fig. S7).

Moreover, the monolayer generated in the “in-3D” culture on the nanograss was accompanied by cell height increase and cell sheet buckling (Fig. 4b), which presumably reflected the remaining restrictive effect of substrate nanotopography, which was not counterbalanced by Matrigel supplementation. These findings agree with the predictions of the previously proposed hypothesis on cell confinement and buckling - when an epithelium is confined to an area smaller than the area dictated by its mechanical equilibrium, it can relieve the stress either by be homogeneously compressed with cell height increase or buckle^31^,^32^. In addition, cell height increase was also observed in T84 cells on the nanograss (Supplementary Fig. S5). Similar “restricted expansion” induced cell height increase has also been long been proposed to be involved in the lens placode formation^27^,^28^, in which matrix-mediated adhesion between the ectoderm and the underlying optic vesicle restricted the expansion of the ectoderm and continued cell proliferation results in thickening of the head ectoderm to form the placode. We propose that substrate nanotopography in the “in-3D” culture, in some regards, may recapitulate the interface between the ectoderm and the optic vesicle and contributed to the cell height increase and cell sheet folding. Because the prevention of cell spreading can cause cell death in some cases^33^,^34^, we speculate that the regain of certain viability of MDCK-II cells on the nanograss in the “in-3D” culture is also due to a partial relaxation of the restriction. However, the remaining restriction is still sufficient to promote spheroids formation of MDCK-II cells. Although MDCK-II cells in the prolonged “in-3D” culture on the flat substrates also formed lumens, these lumens were generated by epithelium buckling and wrapping under the condition of high confluency of cells (Fig. 3d). This difference in the mechanism of lumen formation further explained why MDCK-II featured different polarities in the “in-3D” culture on the nanograss and flat substrates.

The Calu-3 cell line is a well-differentiated and characterized epithelial cell line, derived from metastatic site of human bronchial submucosal gland^35^. Previous report has shown that several other bronchial epithelial cell types could also develop into spheroids without lumens in the 3D Matrigel culture^36^. Calu-3 cells cultured on the nanograss without Matrigel supplement in the present study displayed two major morphologies: spheroids with lumens, and without lumens (Fig. 2), of which showed positive staining of caspase-3 (Supplementary Fig. S2). Whether all those concrete spheroids will finally develop a hollow lumen is unclear. We further attempted to reverse the polarity of the Calu-3 spheroids generated on the nanograss using a Matrigel overlay assay which was previously used to reverse the polarity of MDCK cysts formed in suspension culture^17^. A little unexpectedly, the polarity of the spheroids remained unchanged after 3 day Matrigel overlay (Supplementary Fig. S9). Considering that the “in-3D” culture also did not induce Calu-3 lumen formation on the flat substrates, we are not clear about the exact role of the chemical cues provided by Matrigel in the lumen formation and polarity establishment for Calu-3 cells.

**Figure 5:**
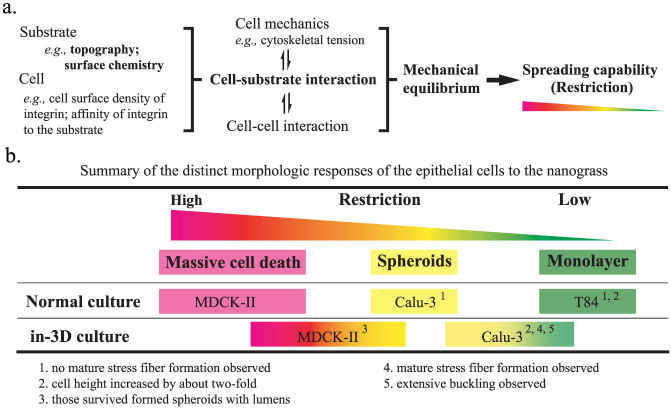
Proposed underlying mechanisms for cell restriction and distinct morphologic responses of MDCK-II, Calu-3 and T-84 cells to the nanograss. (a) Proposed factors that determine the mechanical equilibrium and spreading capability of cell. (b) Summary of cell morphologic responses to the nanograss. Different colors represent different level of sensitivity of the cell to the confinement of the nanograss.

Putting all pieces together, we considered a spectrum of the distinct responses of MDCK-II (and BEAS-2B and HCT116), Calu-3 and T84 cells to the nanograss as a result of inherent, cell-type specific mechanical equilibrium of the cell and capability of cell spreading, which is determined by cell mechanics, cell-cell interaction and especially, in our case, cell-substrate interaction (Fig. 5a). The cell-substrate interaction varies with cell type, substrate topography and substrate surface chemistry. Reduced adhesion sites and intense, dynamic and reciprocal cell-substrate interaction at the cell-nanograss interface confined cells of different types to different states of mechanical equilibrium, of which only some allow cells to survive and proliferate. In normal culture, MDCK-II, BEAS-2B and HCT116 cells were highly sensitive to substrate nanotopography and thus under high restrictive effect, which led to massive death on the nanograss. Calu-3 cells are less sensitive to substrate nanotopography, such that they could survive and respond with different morphologic changes, namely lumen formation. T84 cells are the least sensitive to substrate nanotopography and therefore form monolayers. However, in the “in-3D” culture, the surface chemistry of the nanograss was modified by Matrigel coating, which led to a partial relaxation of the restriction and therefore a shift of cell response (Fig. 5b).

Various material-engineering tools at the nano and micro scales have been employed to induce tissue-like morphogenesis of cells, particularly in the case of endothelial and epithelial cells that can form lumens^9^,^10^,^33^,^37^,^38^,^39^. However, most of these approaches involved partially combining micro and nano patterning with the use of 3D-gel cultures in which Matrigel is often used (Supplementary Fig. S8, Geometry circle and 3D gel circle). Therefore, in previous studies, the effect of substrate topography on organoid formation might be masked by the effect of Matrigel. The results that we have presented here demonstrate that substrate nanotopography alone, as the single initial physical cue, is sufficient to induce similar epithelial tissue-like morphogenesis (Supplementary Fig. S8, Topography circle).

Recently, increasing attention has been devoted once again to analyzing the mechanical cues that govern the generation of multicellular shapes during embryonic development and tissue morphogenesis^40^. Although a lot of other artificial micro and nano-structured surfaces like groove and pillar patterns have been introduced to study their effects on cell behavior^41^,^42^, the nanograss induction of epithelial organoids formation we demonstrated - probably arising from the uniqueness in nanograss isotropy and flexibility - has highlighted that substrate topography plays a critical role in dictating cell fate and morphogenesis. Further investigation is required to uncover the mechanical and biochemical mechanisms that underlie the process by which single Calu-3 and MDCK-II cells sense and interpret topographic cues at the molecular level to coordinate cellular and multicellular morphologic behaviors such as cell height increase, spheroids formation and cell sheet folding. Our results also raise the possibility that the effect of other substrate topographic features on the tissue-like morphogenesis of epithelial cells can be evaluated in an effort to engineer complex tissue structures such as tubular networks. Future tissue-engineering systems could be effectively developed by integrating carefully designed substrate topography with substrate-geometry engineering and 3D culture assays performed using microfluidic platforms^3^,^43^,^44^. Since abnormal physical and biomechanical changes in ECM also always accompany disease condition such as tumor progression^45^,^46^, future work should also be devoted to explore the causative role of ECM topography in cancer pathogenesis.

## Methods

### Fabrication and preparation of nanograss-patterned silicon substrates

Randomly aligned nanograss was fabricated on silicon substrate by using a modified Bosch deep-reactive ion etching (DRIE) process as previously described^47^. Briefly, we carefully modified a conventional DIRE process involving cyclic passivation and etching modes that is used for creating structures featuring high aspect ratios. The ratio of the etching and passivation duration was adjusted to prevent the passivating polymer film from being completely removed, which allowed a random nanomask to be obtained using the residual polymer particles. Next, additional cycles of etching and passivation gradually created the nanograss feature on the silicon substrate. A 4-inch wafer sample was then diced into chips sized 1 × 1 cm^2^ that were used in the cell-culture experiments. Before culturing cells, all substrates were cleaned using acetone and then isopropanol in an ultrasonic bath for 3 min. After carefully rinsing the samples in DI water and blow-drying, the samples were treated with O_2_ plasma for 3 min to render the surface hydrophilic, and the chips were then sterilized in 70% ethanol for 15 min and washed 3 times with phosphate buffered saline (PBS). The sterilized substrates were incubated with cell-culture medium at 37°C for 15 min before seeding cells.

### Cell culture

Human-airway epithelial Calu-3 cells (ATCC® HTB55™) were cultured in Eagle's minimum essential medium (MEM, Gibco) and 1% sodium pyruvate; MDCK II cells (Madin-Darby canine-kidney cells, ECACC 00062107) and T84 cells (ATCC® CCL-248™) in Dulbecco's Modified Eagle Medium: Nutrient Mixture F-12 (DMEM/F-12, Invitrogen); and BEAS-2B cells (ATCC® CRL-9609™) and HCT116 cells (ATCC® CCL-247™) in Dulbecco's modified Eagle's medium (DMEM, Invitrogen). All media were supplemented with 10% FBS. All cell cultures were maintained in a 95% air/5% CO_2_ atmosphere at 37°C. Cells were passaged at a 1:3 dilution after they were approximately 70% confluent, and then the culture medium was changed once every 3 d. To culture cells on nanograss-patterned and flat substrates, cells were seeded at a density of approximately 1 × 10^4^ cm^−2^ on each sample, which was then placed in a 35-mm polystyrene tissue-culture dish. To perform “in-3D” culture assays, culture medium was first cooled to 4°C before mixing with 2% Matrigel (Corning) and subsequent mixing with cells.

### Cell viability assays

The viability of adherent cells on each substrate was quantified by concurrently staining viable and dead cells with the fluorescent dyes Calcein-AM (Life Technologies, Inc., NY) and propidium iodide (PI, Sigma-Aldrich), respectively. Adherent cells on substrates were first gently washed with PBS and then incubated with a mixture of cell-culture medium, Calcein-AM (2 μg/ml), and PI (50 μg/ml) for 20 min at 37°C to allow the dyes to be absorbed, after which epifluorescence imaging was performed. Images were collected from 5 random fields by using the 10× lens of an epifluorescence microscope (FN1; Nikon, Japan). The numbers of alive (stained green) and dead (stained red) adherent cells were determined from these images, and the death rates (ratios of dead to total cells) were pooled.

### Immunostaining and confocal microscopy

Briefly, cells were fixed and permeabilized using, respectively, 4% paraformaldehyde in PBS and 0.2 M NH_4_Cl/PBS containing 0.2% Triton X-100 for 10 min at room temperature. After blocking with 3% bovine serum albumin (BSA; Sigma-Aldrich) in PBS for 2 h, F-actin and the nuclei were stained with Phalloidin-TRITC (2 μg/mL; Sigma-Aldrich) and DAPI (1 μg/mL; Sigma-Aldrich) for 60 min at room temperature, respectively. To characterize Calu-3 spheroid polarity, cells were first incubated with rabbit polyclonal anti-ZO-1 (1:100 dilution; Zymed Laboratories Inc.) and mouse monoclonal anti-integrin β1 antibodies (1:500 dilution; Abcam) overnight at 4°C. To identify apoptosis within Calu-3 spheroids, cells were first incubated with rabbit polyclonal active Caspase-3 (1:200 dilution; Abcam) overnight at 4°C. After washing with PBS 3 times (5 min each), the cells were incubated with FITC-conjugated donkey anti-rabbit IgG and TRITC-conjugated donkey anti-mouse IgG (both 1:200 dilution; Jackson ImmunoResearch) for 1 h to detect ZO-1, active Caspase-3 and β1 integrin, respectively. Samples were lastly washed 3 times with PBS, followed by examination under a confocal microscope (LSM710, Zeiss, Germany).

### Quantification of the area of cell-colony projection

On Days 1, 3, and 6, confocal images of 10 random fields were collected using a 10× lens; the focus was set at the cell-substrate interface. The area of cell-colony projection was quantified using ImageJ software (NIH).

### Scanning electron microscopy

Nanograss patterns and cell morphologies were verified using SEM analysis (JSM-6700F and JSM-7100F, JEOL, Japan). Cells were fixed with 2.5% glutaraldehyde for 1 h at room temperature, rinsed 3 times with PBS, and dehydrated using a graded ethanol series (30%, 50%, 70%, 80%, 90% v/v ethanol in ddH2O, 10 min each). The samples were then immersed in 100% ethanol and critical-point drying (CPD) was performed using a CPD-2 instrument (Pelco TM). After the samples were dry, they were mounted on aluminum stubs and sputter-coated with gold and then used in SEM examination.

### Statistics

All data are expressed as means ± S.E. Statistical analysis was performed using unpaired, 2-tailed Student's *t* tests. Significance levels were set as follows: statistically significant (0.01 ≤ *P < 0.05), highly significant (0.001 ≤ **P < 0.01), and extremely significant (***P < 0.001).

## Author Contributions

Y.S., P.H. and L.Y. conceived the original concept. Y.S. designed and executed most of the experiments. Y.H. and S.Y. optimized the process recipe and fabricated the substrates. Y.S., P.H. and L.Y. analyzed the data and, with comments from Y.H. and S.Y., wrote the manuscript. P.H. and L.Y. supervised the study.

## Supplementary Material

Supplementary Information Supplementary Information

Supplementary Information Supplementary Video
