# Supplementary material for: In Vitro Epithelial Organoid Generation Induced by Substrate Nanotopography
Source: Sci Rep. 2015 Mar 19;5:9293. doi: 10.1038/srep09293 (PMC4365384; doi:10.1038/srep09293)
Supplement: Supplementary Information [file srep09293-s1.pdf]

In Vitro Epithelial Organoid Generation Induced by Substrate Nanotopography

Yusheng Shen[1], Youmin Hou[2], Shuhuai Yao[2], Pingbo Huang[1, 3, 4]*, and Levent Yobas[5]*

[1]Division of Biomedical Engineering, [2]Department of Mechanical Engineering, [3]Division of Life Science, [4]the Sate Key Laboratory of Molecular Neuroscience, [5]Department of Electronic & Computer Engineering, The Hong Kong University of Science & Technology, Hong Kong. Correspondence and requests for materials should be addressed to P. Huang (email: bohuangp@ust.hk) and L. Yobas (email: eelyobas@ust.hk).

Supporting Information

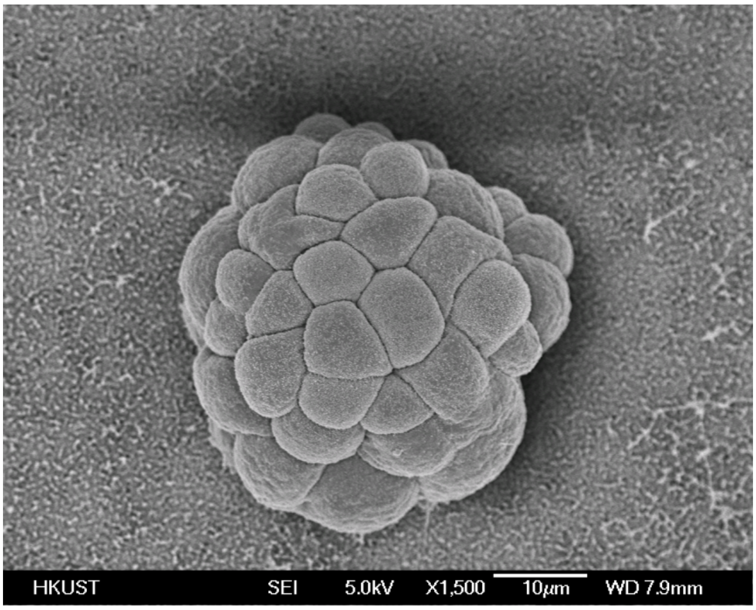

Supplementary Figure 1. SEM image of a Calu-3 spheroid after culturing for 6 d on the nanograss-patterned substrate. Scale bar, 10 μm.

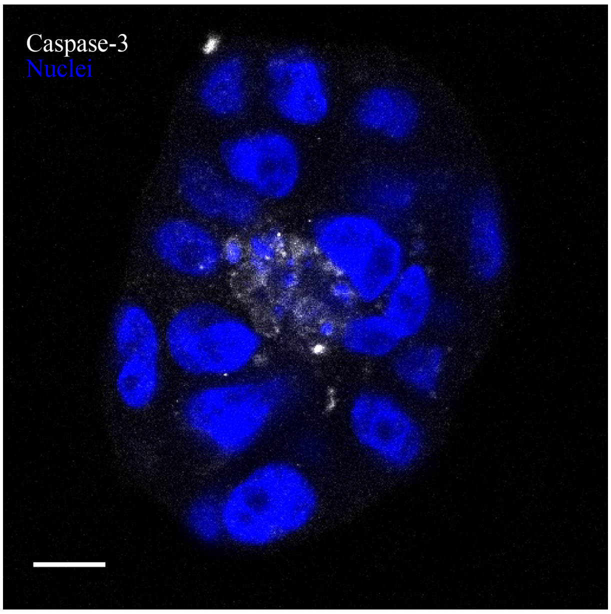

Supplementary Figure 2. Active Caspase-3 (white) was present in the center of the Calu-3 spheroids on Day 6, accompanied with nuclear fragmentation. Scale bar, 10 μm.

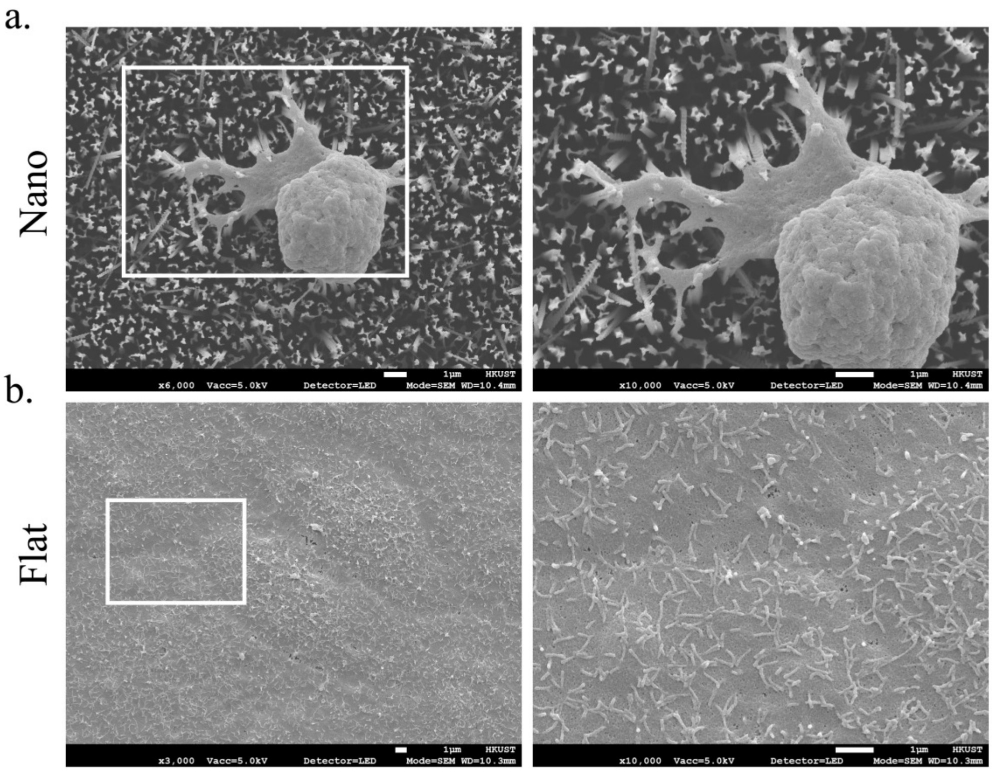

Supplementary Figure 3. SEM images of MDCK-II cells cultured on nanograss-patterned and flat substrates for 12 h and 3 d, respectively. (a) On the nanograss-patterned substrate, dead MDCK cells appeared round and shrunken and interacted extensively with the nanograss at the interface, which is indicated by the substantial bending of nanospikes toward the cell. Microvilli were absent on the surface of cells cultured on the nanograss-patterned substrate, but abundant on the

apical surface of MDCK-II cells cultured on the flat substrate (b). Together with the confocal microscopy results in Figure 3, these observations suggest that the cell membrane was no longer intact after MDCK-II cells adhered to the nanograss. Images on the right side in (a) and (b) are the insets of the images on the left. Scale bars in left-side images and insets, 1 μm.

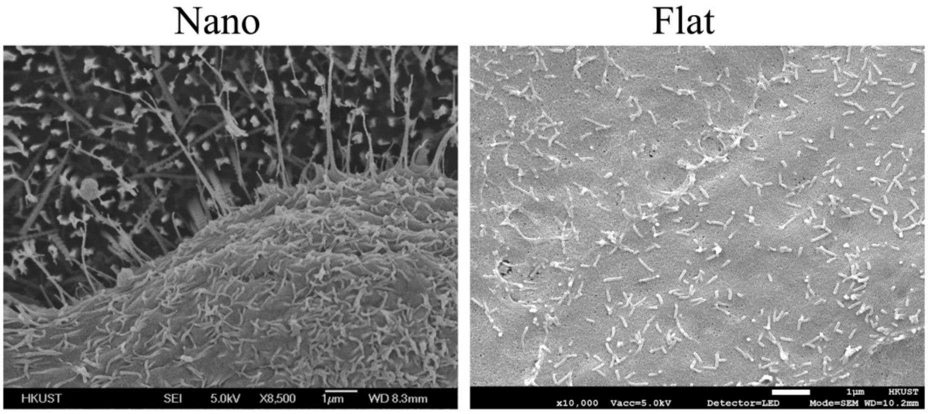

Supplementary Figure 4. SEM images of Calu-3 cells cultured on the nanograss-patterned and flat substrates for 1 and 6 d, respectively. Scale bar, 1 μm.

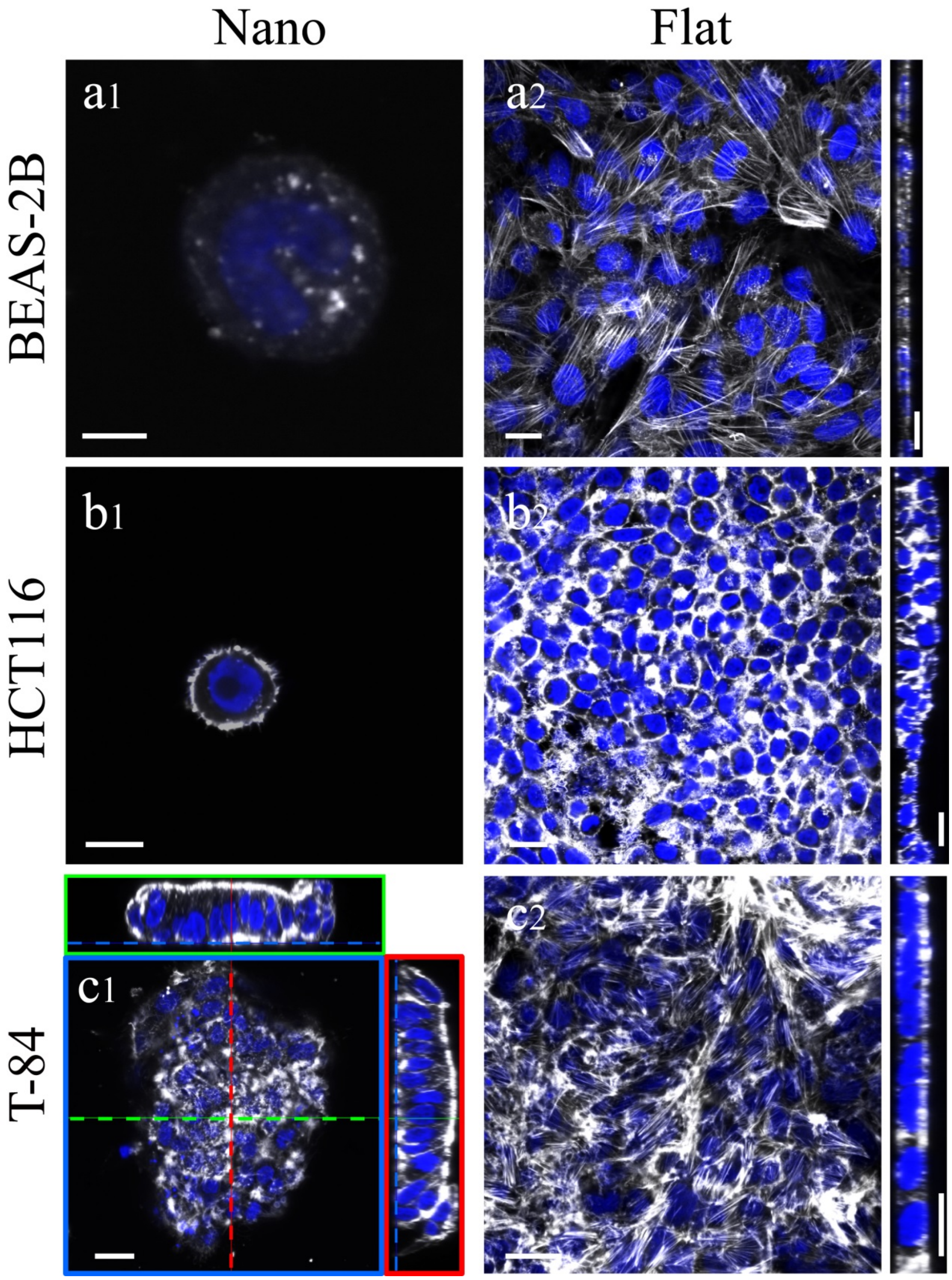

Supplementary Figure 5. BEAS-2B and HCT116 cells (a1, b1) encountered massive cell death on the nanograss while T84 cells (c1, c2) formed monolayer colonies and confluent monolayer on the nanograss and the flat substrates, respectively. The cell height of T-84 cells cultured on the nanograss (about 30 μm) was much higher than that on the flat substrates (about 10 μm) with the distribution of F-actin disorganized (c1) and fiber-like (c2). F-actin, white; nuclei, blue. Scale bars, 5 μm (a1, b1); 20 μm (a2, b2, c1, c2).

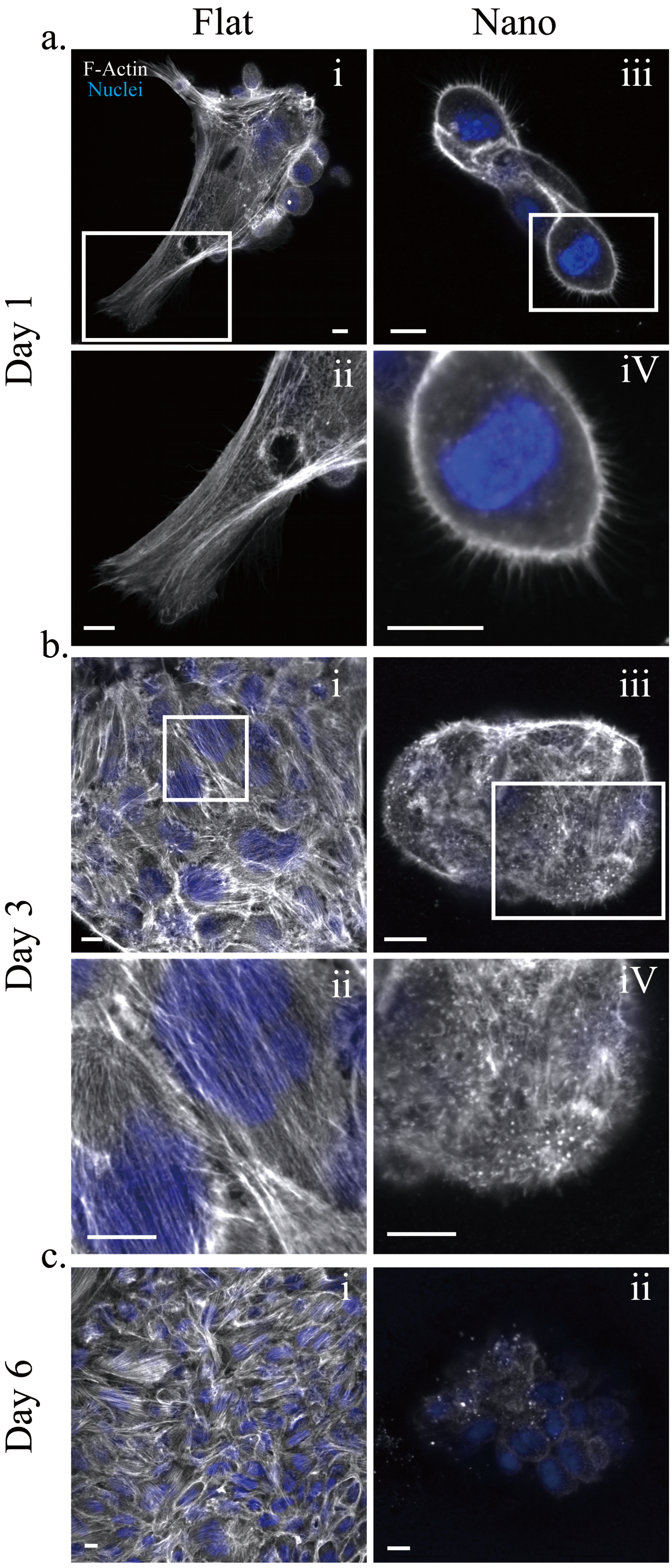
Flat
Nano
a.
F-Actin
Nuclei
i
iii
ii
iV
Day 1
b.
i
iii
ii
iV
Day 3
c.
i
ii
Day 6

Supplementary Figure 6. Calu-3 cells failed to form lamellipodia and mature stress fibers on the nanograss-patterned substrate. Confocal images of representative Calu-3-cell colonies on Days 1 (a), 3 (b), and 6 (c) are shown with co-staining of F-actin (white) and the nuclei (blue). The X-Y planes are focused at the cell-substrate interface. Scale bars, 10 μm.

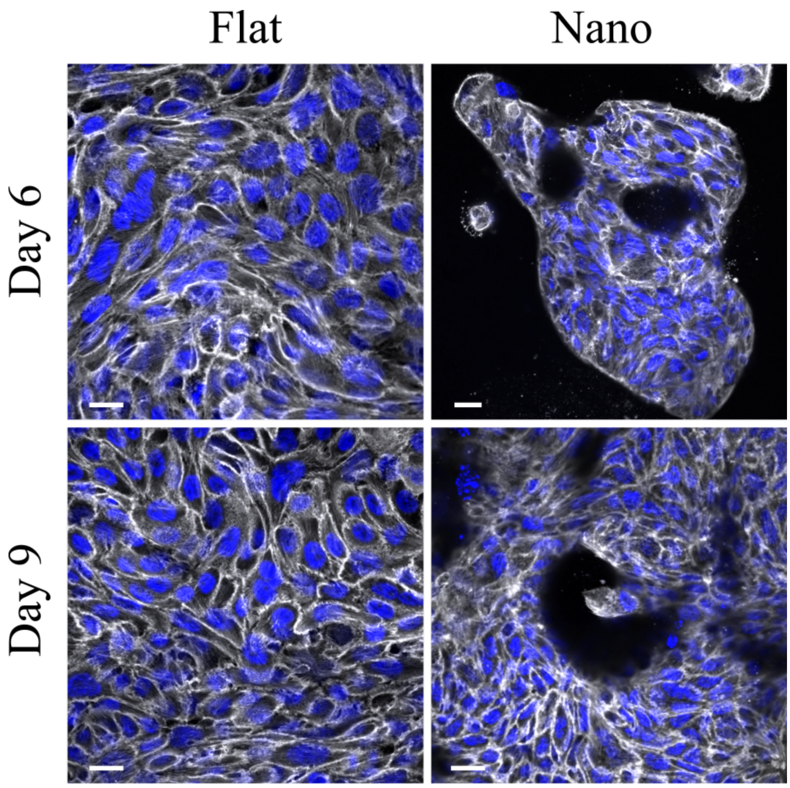

Supplementary Figure 7. Calu-3 cells formed stress fibers on the nanograss in the "in-3D" culture. F-actin, white; nuclei, blue. Scale bars, 20 μm.

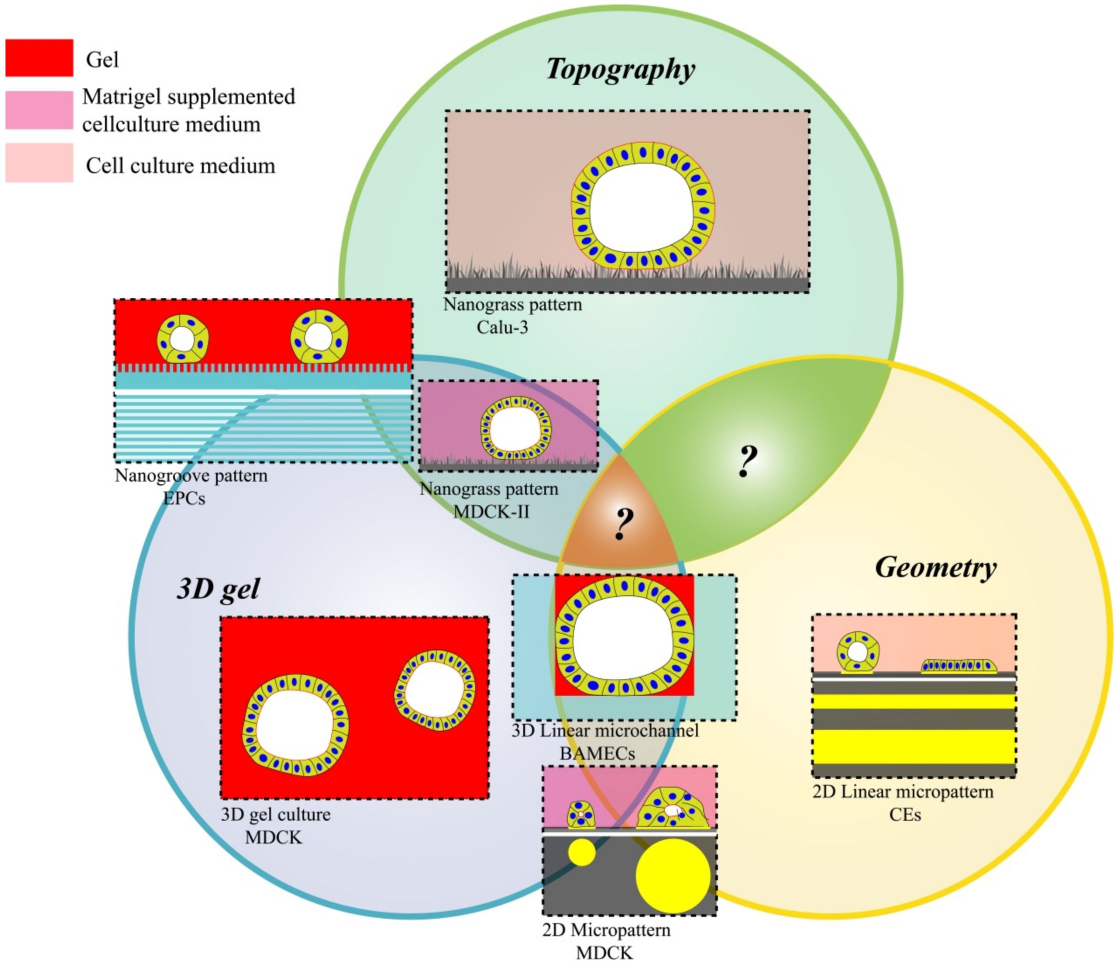

Supplementary Figure 8. A summary of 3 major material-engineering methods at the micro and nano scales for controlling tissue-like morphogenesis of cells. Capillary-tube (lumen) formation by endothelial cells could be geometrically controlled by micropatterning cells onto linear patterns (yellow lines in the yellow Geometry circle)[1]. Large linear patterns that poorly restrict cell growth and spreading cannot induce the formation of capillary structures. After the advent and wide use of 3D-gel cultures, mature cysts that were formed became the optimal culture model for investigating the molecular mechanisms underlying epithelial morphogenesis (blue 3D-gel circle)[2, 3]. These techniques engendered the combination of substrate patterning (at both the nano and micro scales) with 3D cultures (circle intersections). Microchannels (3D geometry) and micro patterns (2D geometry) used together with 3D cultures induced the formation of capillaries and cysts[4, 5]. The use of a nanogroove pattern (topography) together with Matrigel cultures was demonstrated to enhance endothelial-cell capillary-tube formation[6]. However, these previous studies did not examine the effect of substrate topography alone on tissue-like morphogenesis of cells, and this strongly motivated us to investigate how epithelial cells respond to nanotopographic features (illustrated in the green Topography circle).

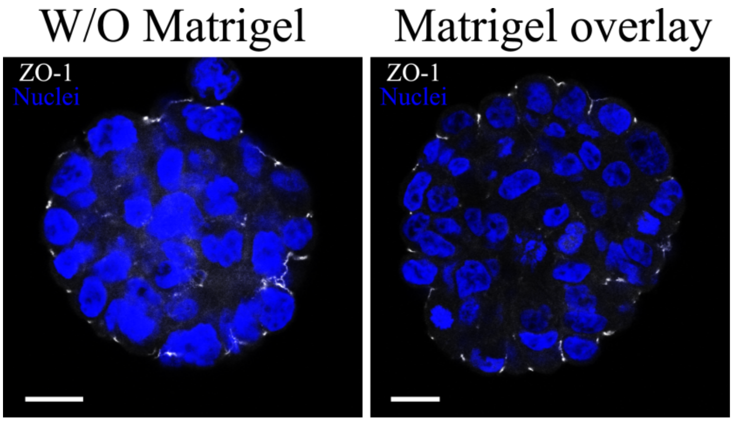

Supplementary Figure 9. Matrigel overlay failed to reverse the polarity of the Calu-3 spheroids generated on nanograss. Spheroids were first generated by culturing Calu-3 cells on nanograss for 6 d and then were overlaid with Matrigel for 3 d to induce the reverse of polarity. ZO-1, white; nuclei, blue. Scale bars, 20 μm.

Supplementary Video 1. Calu-3 cells in the "in-3D" culture formed complex structures on the nanograss. This video displayed the whole 3D morphology of the Calu-3 cell colony with the X-Y and Y-Z planes fixed at the focus of interest and the X-Z plane (the moving green line) changing. The white arrowhead indicates the tubular structure. F-actin, white; nuclei, blue.

Supplementary references

1. Dike, L.E. *et al.* Geometric control of switching between growth, apoptosis, and differentiation during angiogenesis using micropatterned substrates. *In Vitro Cell. Dev. Biol. Anim.* 35, 441-448 (1999).
2. Lee, G.Y., Kenny, P.A., Lee, E.H. & Bissell, M.J. Three-dimensional culture models of normal and malignant breast epithelial cells. *Nature Methods* 4, 359-365 (2007).
3. Roignot, J., Peng, X. & Mostov, K. Polarity in mammalian epithelial morphogenesis. *Cold Spring Harb. Perspect. Biol.* 5, a013789 (2013).
4. Raghavan, S., Nelson, C.M., Baranski, J.D., Lim, E. & Chen, C.S. Geometrically controlled endothelial tubulogenesis in micropatterned gels. *Tissue Eng. A* 16, 2255-2263 (2010).
5. Rodríguez-Fraticelli, A.E., Auzan, M., Alonso, M.A., Bornens, M. & Martín-Belmonte, F. Cell confinement controls centrosome positioning and lumen initiation during epithelial morphogenesis. *J. Cell Biol.* 198, 1011-1023 (2012).
6. Bettinger, C.J., Zhang, Z., Gerecht, S., Borenstein, J.T. & Langer, R. Enhancement of in vitro capillary tube formation by substrate nanotopography. *Adv. Mater.* 20, 99-103 (2008).
